# Narrative Review of Mesenchymal Stem Cell Therapy in Renal Diseases: Mechanisms, Clinical Applications, and Future Directions

**DOI:** 10.1155/sci/8658246

**Published:** 2024-12-11

**Authors:** Yanjun Wang, Pengli Luo, Tana Wuren

**Affiliations:** ^1^Research Center for High Altitude Medicine, Qinghai University, Xining 810001, China; ^2^High-Altitude Medicine Key Laboratory of the Ministry of Education, Xining 810001, China; ^3^Qinghai Provincial Key Laboratory for Application of High-Altitude Medicine (Qinghai-Utah Joint Key Laboratory for Plateau Medicine), Xining 810001, China; ^4^Nephrology Department, Affiliated Hospital of Qinghai University, Xining 810001, China

## Abstract

Renal diseases, particularly acute kidney injury (AKI) and chronic kidney disease (CKD), are significant global health challenges. These conditions impair kidney function and can lead to serious complications, including cardiovascular diseases, which further exacerbate the public health burden. Currently, the global AKI mortality rate is alarmingly high (20%–50%); CKD is projected to emerge as a major global health burden by 2040. Existing treatments such as hemodialysis and kidney transplantation have limited effectiveness and are often associated with adverse effects. Mesenchymal stem cells (MSCs) offer considerable potential for treating renal diseases owing to their regenerative and immunomodulatory properties. Thus, this review focuses on the application of MSCs in renal disease, discusses fundamental research findings, and evaluates their application in clinical trials. Moreover, we discuss the impact and safety of MSCs as a therapeutic option and highlight challenges and potential directions for their clinical application. We selected research articles from PubMed published within the last 5 years (from 2019), focusing on high-impact journals and clinical trial data, and included a few key studies predating 2019. Considerations included the novelty of the research, sample size, experimental design, and data reliability. With advancements in single-cell sequencing, CRISPR/Cas9 gene editing, and other cutting-edge technologies, future MSC research will explore combination therapies and personalized treatments to provide more promising, safer treatments with reduced adverse reactions and enhanced therapeutic outcomes. These advances will improve kidney disease treatment methods, enhance patient quality of life, and maximize the benefits of MSC therapies.

## 1. Introduction

Kidney diseases pose a substantial global health threat owing to their rising incidence and mortality rates. These diseases impair kidney function, often leading to other serious complications such as cardiovascular diseases. Acute kidney injury (AKI) and chronic kidney disease (CKD) are the two primary forms of kidney damage, both of which may ultimately lead to multiorgan dysfunction or failure. AKI-related mortality rates are notably high, ranging from 20% to 50% [1]. An analysis focusing on the patterns of renal recovery after AKI indicated that 41.2% of patients with AKI did not regain kidney function before being discharged. Within this subset, the 1-year age-adjusted mortality rate was approximately 60%, considerably exceeding the rate in patients who achieved complete recovery [2]. Moreover, incomplete recover from AKI may lead to persistent kidney damage, thus increasing the risk of developing CKD from AKI. CKD was projected to rise from the 16th position in 2016 to the 5th position by 2040 in the years of life lost rankings [3]. CKD involves a gradual decline in renal function, managed by strategies such as controlling blood pressure and glucose, pharmacological interventions, and, in end-stage cases, renal transplantation, or long-term dialysis. Nonpharmacological strategies, such as lifestyle modifications, including dietary changes, regular exercise, and smoking cessation, are also recommended. However, while pharmacotherapy can slow CKD progression, it cannot reverse renal damage and may cause adverse reactions with prolonged use. Renal transplantation stands as one of the most viable treatments for CKD; however, it is constrained by the availability of donor kidneys and presents a risk of acute and chronic rejection, potentially leading to donor kidney dysfunction and life-threatening complications for the recipient. Immunosuppressive agents used to prevent organ rejection, may also cause infections and other complications. Conventional hemodialysis used in both AKI and CKD, cannot fully substitute all renal functions nor promote the repair of damaged kidneys. Long-term reliance on hemodialysis may lead to vascular access issues and increased infection risk. The progression of AKI is closely linked to immune system activation, where inflammatory responses play a pivotal role in disease development. For instance, the infiltration of inflammatory cells and the release of cytokines can exacerbate renal damage and fibrosis [4]. Current treatments are limited by their inability to substantially reverse renal damage or modulate immune responses.

In this context, mesenchymal stem cells (MSCs), which are known for their self-renewal, differentiation capabilities, and regenerative and immunomodulatory properties [5], have emerged as a promising strategy for treating kidney diseases [6]. For instance, MSCs can alleviate renal inflammation by inhibiting the maturation of dendritic cells (DCs), thereby reducing the expression of CD103-positive DCs in the kidneys [7]. They can also induce an anti-inflammatory phenotype in renal macrophages by transferring their own mitochondria [8]. Moreover, MSCs regulate dysregulated T helper 17 (Th17) and regulatory T (Treg) cells through the mTOR pathway, thereby mitigating renal immune responses [9]. This ability of MSCs to reduce inflammation and immune-mediated renal damage is particularly crucial for AKI and CKD, as these diseases often involve an overactivation of the immune system. Additionally, microRNAs (miRNAs), as posttranscriptional regulatory factors, are involved in the progression of renal fibrosis [10]. MSCs can secrete exosomes containing miRNAs or directly release miRNAs to ameliorate renal fibrosis [11–14]. They also release antifibrotic factors, including hepatocyte growth factor (HGF) and tumor necrosis factor-stimulated gene 6, which play a role in antifibrotic actions [14–16]. This is crucial in cases where poorly recovered AKI progresses to CKD or when CKD advances because of other causes, as renal fibrosis is the underlying pathology of these conditions.

**Figure 1:**
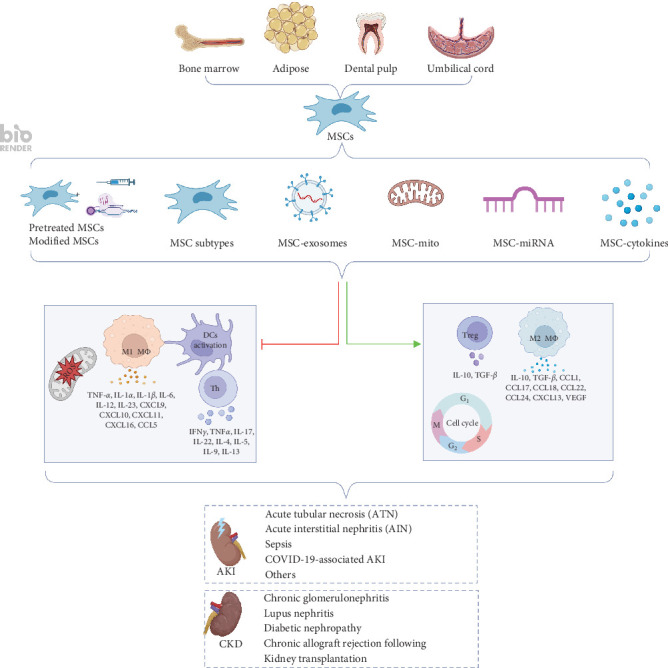
Mechanisms of MSC-mediated immunomodulation and tissue repair in renal diseases. This figure illustrates the roles of MSCs in renal diseases, derived from various tissues, including bone marrow, adipose tissue, dental pulp, and umbilical cord. MSCs can be pre-conditioned or genetically modified, and certain subpopulations may have enhanced therapeutic effects. These cells secrete a variety of bioactive factors, such as exosomes, mitochondria, miRNAs, and cytokines, which play a crucial role in modulating immune responses and promoting tissue repair. MSCs suppress macrophage M1 polarization, Th cell and dendritic cell activation, reduce pro-inflammatory cytokines, and ROS levels, while promoting Treg cell expansion and macrophage M2 polarization, thus regulating the cell cycle. These mechanisms collectively contribute to the repair of both acute and chronic kidney diseases.

The purpose of this review was to explore the basic research and clinical trials involving MSCs in kidney therapy, analyze their therapeutic mechanisms, and discuss the potential challenges associated with their clinical application. We also discuss the applications of MSCs in clinical trials, including their safety, impact, and patient outcomes.  Figure 1 illustrates the various tissue origins of MSCs, the secretory factors they produce, and their roles in immunomodulation and tissue repair within the context of renal diseases.

## 2. Basic Characteristics of MSCs

MSCs, a type of pluripotent stem cell, are distinguished by their ability to adhere to and grow on surfaces during in vitro culture. These cells are identified by the presence of CD90, CD73, and CD105 expression and the absence of CD34, CD45, CD19, CD11a, and human leukocyte antigen-DR expression [17]. MSCs are now widely considered one of the most promising cell types in cell therapy for the following reasons: (1) MSCs are abundant and can be isolated from various tissue sources, such as Wharton's jelly of the umbilical cord, bone marrow, adipose tissue, and dental pulp; (2) they exhibit low immunogenicity, expressing major histocompatibility complex (MHC) class I molecules but not MHC class II molecules; (3) tissues from which MSCs are derived are easy to collect, involve less ethical controversy and constraints, and can be cultured and subcultured in vitro (e.g., MSCs derived from the umbilical cord, considered medical waste, involve less ethical controversy); (4) MSCs can differentiate into multiple cell types in vitro, but their primary mechanism of action in clinical settings involves the secretion of paracrine and autocrine cytokines and mitochondrial transfer, which aid in the regulation and repair of damaged target cells [11, 18, 19]; (5) MSCs can regulate the function of key immune cells in the body, including antigen-presenting cells, T cells, B cells, and natural killer cells, thereby influencing critical immune functions such as antigen recognition and elimination [20]. These characteristics make MSCs highly promising for applications in cell and tissue repair and regenerative medicine [21].

## 3. Identification and Functional Specificity of MSC Subpopulations

Previous basic experiments and a series of recent single-cell RNA sequencing (scRNA-seq) studies have revealed substantial cellular heterogeneity within MSCs [22, 23]. ScRNA-seq is an advanced genomic technique that enables the extraction and quantification of RNA molecules from individual cells to precisely analyze their gene expression profiles. This technology provides an unprecedented level of detail in understanding cellular functions and complex biological systems by uncovering unique transcriptional activities in cells at different developmental stages, tissue contexts, or disease conditions. Through scRNA-seq, researchers have successfully elucidated the transcriptional characteristics of individual MSCs, revealing distinct gene expression patterns that define subgroups with various functions and therapeutic potentials. Different MSC subpopulations may possess varied regenerative and immunomodulatory properties that should be considered in future clinical applications. For instance, scRNA-seq has revealed that umbilical cord-derived MSCs (UC-MSCs), particularly those derived from Wharton's jelly (WJ-MSCs), express lower levels of inflammatory factors and higher levels of anti-inflammatory factors than MSCs derived from the bone marrow and adipose tissue. Moreover, WJ-MSCs lack antigen processing and presentation capabilities, making them more advantageous for immune suppression and antiaging applications [24]. Further scRNA-seq studies have identified several subpopulations of WJ-MSCs. These include proliferative MSCs (high proliferative potential), niche-supporting MSCs (rich in extracellular matrix [ECM]-related molecules), metabolism-associated MSCs (related to metabolic capacity), and biologically functional MSCs (regenerative and immunomodulatory functions). Notably, biomolecules related to angiogenesis and wound repair are substantially enriched in the umbilical cord segments closer to the fetus, whereas ECM-related molecules are more prevalent near the maternal segments [25]. Another scRNA-seq study found that UC-MSCs exhibit enhanced cellular growth, reduced aging, and a more diverse range of stem cell characteristics [26]. In bone marrow-derived MSCs (BM-MSCs), distinct subpopulations have been identified that align with osteogenic, chondrogenic, and adipogenic pathways. These analyses further indicate that the osteogenic pathway is predominantly active during the initial phases of BM-MSC development. As development progresses, there is a shift toward the prevalence of cells undergoing adipogenic differentiation, alongside those in a quiescent state [27]. Additionally, MSCs from CKD rats exhibit characteristics of premature aging and a loss of regenerative potential [28], suggesting that the quality of autologous MSCs should be considered when using them to treat renal diseases. Furthermore, another single-cell sequencing study [29] demonstrated that the C1 subpopulation of human UC-MSCs belongs to an immunosuppressive subgroup defective in regulating B-cell functions, thus showing no significant impact in lupus mice.

These differences in MSC subpopulations may account for the inconsistent and variable outcomes observed in some clinical trials involving MSCs. Understanding the functional specificity of these subgroups is crucial for optimizing their clinical application. This knowledge can assist researchers and clinicians in identifying subgroups most likely to positively impact specific disease states, facilitating the selection of the most suitable MSC subpopulations for specific therapeutic purposes. For instance, recent clinical trials involving MSCs have consistently avoided selecting senescent MSCs and those from donors with impaired immune functions. During UC-MSC extraction, it has been observed that cells from younger donors are smaller, exhibit stronger proliferation capabilities, and are less prone to senescence during passaging compared to those from older donors [30]. This emphasizes the importance of using standardized and functionally validated MSC preparations in preclinical and clinical trials.

With advances in single-cell technology, future research may continue to uncover new MSC subpopulations and their specific roles, thereby enhancing the effectiveness of existing cell therapies and accelerating the development of new therapeutic strategies to treat currently incurable diseases. Future strategies should focus on selecting MSC subpopulations with antiaging, proangiogenic, and antifibrotic functions not only for treating CKD, but also for other conditions where these properties are beneficial. Additionally, selecting subpopulations with enhanced immunomodulatory and cell-proliferative properties could be crucial for treating AKI, steroid-resistant glomerulonephritis, and a wider range of inflammatory and degenerative diseases. Therefore, an improved understanding of the cellular, molecular, and functional characteristics of MSCs will facilitate more effective utilization of their therapeutic potential.

## 4. Preclinical Insights on MSCs in Renal Disease

An increasing body of evidence supports the therapeutic potential of MSCs in treating renal and other diseases. Multiple in vitro and in vivo studies have confirmed the therapeutic effects of MSCs in treating AKI and CKD, involving various mechanisms.

### 4.1. Application of MSCs in the Treatment of AKI

Tubular epithelial cells (TECs) are the primary target cells in AKI; they play a crucial role in the innate immunity of the kidneys because of their extensive expression of toll-like receptors [31, 32]. However, the specific roles of TECs in kidney protection and repair in response to immunomodulatory factors secreted by MSCs and the complex mechanisms underlying these processes require further in-depth research. MSCs can considerably ameliorate AKI by modulating the balance between Th17 and Treg cells [9]. Additionally, miRNA sequencing has identified several key miRNAs in MSC-derived exosomes from human umbilical cords, such as miR-125 b-5 p, which promotes tubular repair by reducing cell-cycle arrest and TEC apoptosis through the regulation of the p53 signaling pathway. These exosomes localize in ischemic kidneys and concentrate in the proximal tubules, thereby alleviating renal ischemia/reperfusion injury in mice [33]. Beyond the single-cell analysis of MSCs, researchers have employed scRNA-seq to compare the kidneys of AKI mice treated with UC-MSCs with those of untreated mice [34]. The study revealed transcriptional diversity in TECs and immune cells during AKI, emphasizing the importance of multifaceted treatment strategies. The study also showed that MSCs can activate the reparative properties of renal progenitor/stem cells, suppress proinflammatory cytokine expression, interrupt intercellular crosstalk, inhibit proinflammatory monocyte infiltration, and block Th17 cell infiltration to alleviate renal fibrosis, ultimately reducing kidney injury. These findings demonstrate the potential of MSCs in the treatment of AKI.

The therapeutic effects of MSCs in AKI have been enhanced through pretreatment methods. For instance, angiotensin-converting enzyme 2 (ACE2) is highly expressed in renal TECs and degrades angiotensin II (Ang II) into angiotensin (Ang)- (1–7), thereby mitigating the adverse effects of Ang II. Ang- (1–7) exerts anti-inflammatory, antiapoptotic, and antioxidative stress effects by acting on Mas receptors. Researchers have enhanced the anti-inflammatory, antioxidative stress, and antiapoptotic effects of MSCs by overexpressing ACE2, which provides additional protection against ischemia/reperfusion kidney injury in both in vitro and in vivo models [35]. Additionally, the selective dopamine D1 receptor agonist fenoldopam mesylate improves the survival of MSCs under oxidative stress, thereby enhancing their therapeutic effect on AKI [36]. Electrical field stimulation enhances the localization of MSCs, thereby improving their therapeutic potential against acute cisplatin nephrotoxicity [37]. Furthermore, the development of an MSC system for nitric oxide delivery facilitates AKI therapy by promoting angiogenesis, stimulating cell proliferation, and exerting anti-inflammatory effects through macrophage polarization [38].

Understanding the functional heterogeneity of MSCs, particularly in terms of immunomodulation and cell trafficking, is crucial for optimizing their application in AKI treatment.

### 4.2. Application of MSCs in the Treatment of CKD

Common causes of CKD include chronic nephritis, diabetic kidney disease (DKD), and lupus nephritis (LN), which involve various factors such as autoimmune responses, metabolic disorders, inflammation, and glomerular injury. Ischemia and fibrosis are major contributors to the progression of CKD, leading to irreversible damage and functional decline of renal tissues, which complicates treatment and delays observable therapeutic outcomes, posing challenges in the design and interpretation of CKD treatment strategies. Recent research has illustrated the capacity of MSCs to promote angiogenesis, For instance, studies on MSCs in ischemic conditions demonstrate their ability to secrete various proangiogenic factors that enhance vascularization and improve tissue perfusion [39]. This capability of MSCs to promote blood vessel formation further underscores their therapeutic potential in treating CKD. Similarly, BM-MSC exosomes, rich in vascular endothelial growth factor (VEGF), reduce transforming growth factor-beta (TGF-*β*) expression [40], and UC-MSCs have also been found to reduce TGF-*β* levels [41]. The modulation of TGF-*β*, a central mediator of fibrosis, illustrates the multifaceted mechanisms by which MSCs exert their therapeutic effects in CKD.

The ability of MSCs to enhance vascularization and reduce fibrosis highlights their broad therapeutic potential, which becomes even more critical when addressing the growing burden of DKD as a leading cause of CKD. Over 40% of diabetic patients progress to CKD, underscoring the need for further research on MSC therapy for DKD [42]. In vitro and in vivo studies have shown promising results for MSC-based therapies in DKD. For instance, BM-MSCs transfected with miR124a have been shown to reduce podocyte caspase-3 expression, decrease apoptosis, and improve renal function by inhibiting the Notch pathway [43]. This highlights the critical role of gene-modified MSCs in modulating pathways central to CKD progression, offering a dual approach that involves both protective mechanisms and regenerative outcomes. Another study using scRNA-seq technology demonstrated that miR-146a-5 p from UC-MSCs promotes M2 macrophage polarization by downregulating the TRAF6/STAT1 pathway, thereby enhancing the anti-inflammatory and antifibrotic effects of MSCs in DKD [14]. These studies provide a theoretical foundation for miRNA-modified MSC therapy for DKD. Research on improving DKD-related renal fibrosis has shown that adipose-derived MSC (AD-MSC) exosomes can reduce TGF-*β* expression by inhibiting the overactivation of the Smad1/mTOR pathway [44]. In addition to gene—and miRNA-modified MSC approaches, other mechanisms also play a significant role in MSC-based therapies for DKD. Several studies have confirmed that MSCs can promote endothelial nitric oxide synthase phosphorylation [45], improve mitochondrial function [46], and reduce reactive oxygen species production, significantly inhibiting oxidative stress and thereby improving renal function. These mechanisms are particularly relevant for DKD, where oxidative stress dysregulation is a key factor in disease progression. Moreover, MSCs have demonstrated promising results across different intervention types. A review of syngeneic, autologous, allogeneic, and xenogeneic cell-based interventions highlights the effectiveness of MSCs in alleviating DKD. For instance, allogeneic MSCs can effectively reduce inflammation and fibrosis, while syngeneic autologous BM-MSCs can reduce immune inflammation and prolong kidney graft survival in patients with DKD [47]. Another meta-analysis of cell-based interventions for DKD [48] indicates that MSCs derived from bone marrow and umbilical cord/amniotic fluid outperform AD-MSCs in enhancing kidney function and reducing proteinuria.

Other causes of CKD, such as chronic nephritis, are primarily mediated by humoral immunity, while LN is a severe complication arising from the autoimmune disease systemic lupus erythematosus (SLE). Current treatments for renal diseases, such as immunosuppressive therapy, often lead to generalized immune suppression and increased risk of infection. In contrast, MSCs can selectively modulate immune responses, potentially reducing these adverse effects while promoting tissue repair. This targeted action highlights how MSCs may address specific limitations of current treatments and provide a more effective approach in clinical settings. Current research indicates that MSCs can inhibit the proliferation of innate and adaptive immune cells, such as natural killer cells, DCs, T cells, and B cells, thereby suppressing renal immune responses. Additionally, MSCs secrete various growth factors, including epidermal growth factor (EGF), HGF, and VEGF, which possess anti-inflammatory and antifibrotic properties [49, 50]. An RNA sequencing study of renal samples from LN mice treated with intraperitoneal injections of human umbilical cord lining MSCs (CL-MSCs) revealed that MSCs mediate immunoregulation. This effect is achieved through a combined mechanism involving chemokines triggered by proinflammatory cytokines and the generation of nitric oxide in macrophages. This dual action resulted in enhanced survival rates and renal function in LN mice [51]. Notably, the quantity of MSCs (cells per mouse body weight) administered via intraperitoneal injections in this study was significantly higher than that observed with intravenous administration. The higher dose was chosen to potentially enhance local cell availability and therapeutic efficacy. While high-dose MSC therapy may exhibit more pronounced effects, the mechanisms of action and safety profile of large-scale MSC delivery through intraperitoneal injections require further investigation and validation.

In summary, previous research has identified various therapeutic mechanisms of MSCs in renal diseases, including anti-inflammatory, antioxidative stress, immunomodulatory, antifibrotic, angiogenic, apoptosis regulatory, and autophagic mechanisms.

## 5. Clinical Advances in MSC Therapy for Renal Diseases

Despite the substantial potential of MSCs in preclinical studies for the treatment of renal diseases in recent years, it is crucial to acknowledge their limitations. Animal models do not always fully replicate human physiological conditions or disease progression. Hence, some of the effects observed in animals may not be reproducible in humans. Thus, the transition from preclinical to clinical research is a critical step toward ensuring the safety and feasibility of MSC therapies in humans. Clinical trial design and regulation, particularly for chronic diseases, can be more complex and require a longer duration, more participants, and stricter oversight. Despite these challenges, the number of clinical trials investigating the application of MSCs in humans is gradually increasing, and preliminary clinical data have demonstrated the potential of MSCs in the treatment of various kidney diseases, including CKD and AKI.

The results of a pilot clinical study conducted in 2010 (NCT00698191) [52] suggested that heterologous BM-MSC infusion in patients with refractory lupus could alleviate lupus activity and maintain stable kidney function. A multicenter prospective clinical study in 2014 (NCT01741857) [53] showed that UC-MSC treatment for active and refractory SLE could improve renal function indicators and reduce the levels of lupus activity markers. Moreover, a phase I clinical trial in 2022 (IRCT2016090729747N1) [54] indicated that AD-MSCs could effectively reduce urinary protein excretion and disease activity in patients with LN. Flow cytometric analysis from a prospective observational study involving peripheral blood from patients with LN treated with UC-MSCs (NCT01741857) revealed immunomodulatory changesn [55]. Specifically, UC-MSCs were found to suppress lupus inflammatory responses by upregulating tolerogenic dendritic cells (CD1c-positive DCs).

Furthermore, an 18-month follow-up single-arm safety study in 2018 (NCT02195323) [56] demonstrated that administering a single dose of autologous MSCs to patients with CKD was safe and well-tolerated, with no statistically significant changes in renal function, robustly affirming the clinical safety of MSC infusion in patients with CKD. A phase Ia dose-escalation clinical trial in 2020 (NCT02266394) [57] revealed that autologous adipose-derived MSC infusion caused increased renal perfusion and estimated glomerular filtration rates (eGFR) in patients with atherosclerotic renovascular disease, along with lower levels of inflammatory markers and systemic blood pressure.

A phase I/II open-label clinical trial in 2019 (NCT01429038) [58] found that early intravenous infusion of BM-MSCs in kidney transplant recipients for longer than 1 year increased Treg cells and improved allograft kidney function. The most extensive study to date on the application of MSCs for the treatment of chronic allograft rejection following kidney transplantation (NCT02563340) [59] was a single-arm, phase I/II study that showed intravenous injection of heterologous BM-MSCs from healthy individuals was safe and, combined with immunosuppressive drugs, could delay the deterioration of transplanted organ function, possibly through the immunomodulatory effects of MSCs on the peripheral B- and T-cell subsets. Conversely, a phase I/II clinical trial of autologous BM-MSCs for chronic kidney transplant rejection in 2022 (NCT03585855) [60] showed that autologous BM-MSC treatment did not result in improved function of the transplanted kidney. This could be attributed to the use of autologous MSCs of inferior quality derived from patients with severe graft dysfunction and long-term immunosuppressant treatment.

Results from an 8-year follow-up randomized controlled trial (RCT) in 2022 (NCT01374854) [61] showed that the combination of UC-MSCs and adult bone marrow mononuclear cell treatment could reduce the incidence of chronic complications such as diabetic nephropathy in individuals with type 1 diabetes. In 2023, a randomized clinical trial on MSCs for DKD (NCT02585622) [62] demonstrated that, compared to a placebo, cell therapy notably reduced progression of eGFR deterioration over an 18-month period.

Furthermore, in a phase II randomized, double-blind, multicenter trial, Swaminathan et al. [63] found that MSCs did not substantially improve renal function or 30-day all-cause mortality in patients with AKI after cardiac surgery (NCT01602328). However, a follow-up study (NCT03015623) [64] showed that MSCs in a bioreactor used in conjunction with hemodialysis improved renal injury markers and inflammatory in patients with AKI. Consecutive studies by Swaminathan et al. also demonstrated that optimizing the treatment modalities of MSCs can considerably enhance their therapeutic outcomes. Furthermore, an ongoing randomized controlled trial, initiated in 2021 (NCT04194671) [65], is investigating the effects of UC-MSCs in patients with severe AKI.

These clinical studies reveal the remarkable potential of MSCs in the treatment of kidney diseases. However, these trials exhibit several common issues that need to be addressed in future research:

1. Sample size and diversity: The sample sizes in these studies are often small, and the diversity of the samples is limited. For instance, many studies involve less than 50 participants, and the patient populations are often homogeneous in terms of ethnicity and comorbid conditions. This can affect the generalizability and reliability of the results. For example, Liang et al. [52] conducted a study with only 15 patients, which limits the statistical power and the applicability of findings to a broader population. Future studies should include larger, more diverse patient cohorts to enhance the external validity of their findings.
2. Study design: Some trials lack randomized controls and sufficient blinding, which can impact the objectivity and accuracy of the outcomes. For instance, single-arm trials, such as the one conducted by Makhlough et al. [56], inherently lack a comparison group, making it difficult to attribute observed effects solely to the MSC treatment rather than other factors. RCTs with proper blinding are crucial to eliminate bias and validate the efficacy of MSC therapies.
3. Follow-up duration: Most studies have relatively short follow-up periods, often ranging from 3 to 12 months, which are insufficient to adequately assess the long-term efficacy and safety of treatments in CKD. Follow-up periods shorter than 12 months are particularly inadequate when evaluating surrogate endpoints such as eGFR decline. Clinical trials suggest that a 30%–40% decline in eGFR over 2–3 years is an appropriate surrogate endpoint for assessing disease progression [66, 67]. Therefore, future studies should include longer follow-up periods to capture the full range of effects, including delayed complications and sustained benefits.
4. Uniformity in assessment standards: The evaluation criteria used in different studies may vary, limiting the comparability of these results. For example, some studies use eGFR as a primary outcome, while others use proteinuria reduction or biomarkers of inflammation. This heterogeneity in outcome measures complicates the synthesis of findings across studies and hinders the development of consistent treatment guidelines. Recent recommendations suggest that standardized outcomes such as eGFR, serum creatinine levels, and patient-reported quality-of-life measures should be used consistently in trials for renal diseases [67, 68].

Future clinical research needs to overcome these shortcomings, further optimize therapeutic strategies, and maximize the improvement of patient quality of life.

## 6. Challenges and Opportunities

Despite the broad prospects of MSCs in the treatment of kidney diseases, several technical, biological, regulatory, and ethical challenges need to be addressed and resolved prior to their successful implementation in clinical practice [69].

### 6.1. Technical Challenges

#### 6.1.1. Cell Preparation and Expansion

MSCs need to be expanded under strictly controlled conditions to maintain their pluripotency and avoid unnecessary early differentiation. Moreover, large-scale production of MSCs is challenging because their proliferative capacity in vitro is limited, and their biological activity may decrease with increasing passage numbers.

#### 6.1.2. Quality Control

Ensuring consistency and safety in MSC products is crucial. This requires precise quality control measures, including the confirmation of cell purity, activity, genotype, and phenotype.

#### 6.1.3. Product Standardization

The clinical application of MSCs requires product standardization, including standardized cell culture conditions, defined cell dosages, delivery methods, and appropriate storage conditions. Currently, these parameters vary greatly across studies and clinical trials, making it difficult to compare and interpret the results. Recent advancements, such as the use of automated systems like CliniMACS Prodigy, a closed and automated cell processing system that ensures standardized Good Manufacturing Practices (GMP)-compliant cell products, have shown promise in enhancing the standardization of MSC isolation and expansion, which is crucial for reducing variability and ensuring GMP compliance [70].

#### 6.1.4. Storage Issues

Long-term storage of MSCs is challenging because they may lose their therapeutic potential. Additionally, freezing and thawing processes may affect cell viability and function. Developing effective cell preservation and transport methods is the key to achieving broad clinical applications of MSCs.

### 6.2. Biological Challenges

#### 6.2.1. Cell Heterogeneity

MSCs are a highly heterogeneous cell population with biological properties and therapeutic potential that may vary substantially between donors and tissue sources. This heterogeneity may affect clinical outcomes, necessitating further research to identify the most suitable MSC subtype for specific applications. Recent advancements in MSC standardization, such as the use of pluripotent stem cell (PSC)-derived MSCs (PSC-MSCs), have been highlighted as potential solutions to obtain homogeneous and genetically uniform MSC populations. PSC-MSCs are derived from a single PSC line, which provides a consistent source of MSCs with potentially faster proliferation and slower senescence. This standardization could help mitigate variability caused by donor differences and isolation methods [71]. To achieve success in clinical MSCs therapies, obtaining a homogeneous population of MSCs is essential. In 2019, the International Society for Cell and Gene Therapy (ISCT) addressed MSC heterogeneity and provided guidelines to reduce variability [5]. The ISCT MSC committee recommended that studies focus on three key aspects: (1) Tissue source origin: MSCs derived from different tissues such as bone marrow and umbilical cord should be specifically labeled as BM-MSCs and UC-MSCs, respectively. This is crucial because MSCs from various tissues display distinct phenotypic and functional properties, as well as differences in their secretome profiles. (2) Stemness properties: These should be demonstrated through both in vitro and in vivo assays. In vitro assays may include clonogenic assays and multilineage differentiation tests, demonstrating the cells' ability to form colonies and differentiate into multiple lineages. Rigorous in vitro testing should involve clonogenic experiments, which are indicative of progenitor status. However, in vivo studies are crucial to show the cells' ability to self-renew and contribute to tissue regeneration. (3) Functional assays: A comprehensive set of functional assays is needed to illustrate the therapeutic mechanisms of MSCs, including i) the secretion of trophic factors (assessing MSCs' ability to support cell growth); ii) immune cell modulation (evaluating how MSCs influence immune cells); and iii) angiogenesis promotion (demonstrating MSCs' capacity to promote new blood vessel formation). These assays should include in vitro and in vivo tests, such as quantitative RNA analyses, flow cytometry, and protein analysis of the secretome.

Furthermore, it is recommended to use licensed MSCs in inflammatory conditions (e.g., treated with IFN-*γ* or TNF-*α*) and compare them with resting MSCs as controls. These properties are essential for MSCs' clinical utility, as evidenced by FDA reviews and recent product approvals, highlighting the importance of comprehensive functional assays. The ISCT also emphasizes the importance of distinguishing between MSCs and mesenchymal stromal cells, as their therapeutic efficacies can differ significantly. MSCs possess self-renewal and multilineage differentiation capabilities, which are critical for regenerative therapies, whereas mesenchymal stromal cells are primarily known for their trophic and immunomodulatory effects. This distinction is crucial for optimizing clinical applications and ensuring the use of the most effective cell populations. Currently, there is no consensus on specific markers for MSC subpopulations. However, certain surface markers of MSCs can be utilized to identify subgroups with distinct biological behaviors. These markers offer a valuable tool for distinguishing the heterogeneity between MSC populations and understanding their diverse functional roles [5, 17, 72–75] (Table 1).

#### 6.2.2. Cell Aging

MSCs can undergo replicative senescence with increasing passages in vitro, which reduces their therapeutic efficacy. The donor's condition also influences the degree of cellular aging and is likely to affect therapeutic efficacy. Addressing this challenge requires the development of new strategies to maintain the youthful state of cells while avoiding uncontrolled cell proliferation. Induced PSC (iPSC)-derived MSCs (iMSCs) provide advantages in mitigating these effects, as they can be genetically modified to minimize cellular aging and maintain a youthful state with high proliferative capacity. Moreover, optimizing initial culture conditions and using xeno-free differentiation protocols can help slow down the senescence process, thus enhancing the cells' regenerative potential [76].

#### 6.2.3. Immune Rejection

Although MSCs have immunomodulatory properties, in rare cases, they may trigger immune rejection responses in the host. Understanding the interactions between MSCs and the host immune system and developing methods to reduce immune rejection are necessary.

#### 6.2.4. Tumor Formation Risk

Although studies have indicated that MSCs have a relatively low risk of tumor formation, this possibility cannot be entirely excluded. Long-term safety monitoring and rigorous clinical trial design are essential to ensure patient safety.

### 6.3. Regulatory and Ethical Issues

#### 6.3.1. Regulatory Guidelines

The clinical application of MSCs must comply with national and international regulatory standards. This includes conducting appropriate preclinical studies before clinical trials and ensuring patient safety and rights are recognized during clinical trials. Developing and adhering to detailed regulatory guidelines are key steps in the clinical translation of MSC treatments.

#### 6.3.2. Ethical Considerations

The use of MSCs raises ethical issues, including cell source choices, patient consent, and privacy protection. All these factors must be considered in the design and implementation of clinical studies involving MSCs. Owing to these factors, UC-MSCs are often preferred in clinical and preclinical research.

#### 6.3.3. Patient Consent

Patients must be fully informed of the potential risks and benefits of MSC treatment before making an informed decision. This requires the medical team to provide comprehensive, accurate, and understandable information and maintain open and honest communication throughout the treatment process.

## 7. Perspectives and Conclusions

With the advancement of single-cell sequencing, CRISPR/Cas9 gene editing, and other cutting-edge technologies, future research on MSCs will delve deeper into exploring combination therapies and personalized treatments to provide more promising, safer treatments with fewer adverse reactions and enhanced therapeutic outcomes.

Personalizing MSC therapies requires tailoring treatment plans based on a comprehensive analysis of the patients' genetic information, genome sequencing, and biochemical and clinical data. For instance, in individuals exhibiting drug resistance, which can arise from various mechanisms such as abnormal drug metabolism, target gene mutations, or alterations in cellular signaling pathways, gene editing technologies can be employed to modify gene expression of MSCs tailored to the patient's specific resistance mechanism. If the resistance is due to abnormalities in a particular cellular signaling pathway, MSCs can be genetically engineered to enhance their regulatory capabilities within that pathway. Additionally, specific kidney diseases such as polycystic kidney disease (PKD), a genetic disorder typically caused by mutations in PKD1 or PKD2 genes leading to the formation of multiple cysts in the kidneys, can be addressed by customizing MSCs to specifically target PKD-related cellular signaling pathways, thereby slowing disease progression. Fabry disease, a genetic metabolic disorder caused by a deficiency of a specific enzyme (*α*-galactosidase A) affecting kidney function, can be approached by engineering MSCs to express the missing enzyme, providing enzyme replacement therapy in vivo to improve kidney function. Furthermore, the behavior of MSCs in vivo, their delivery strategies, and long-term safety still require further investigation. Developing advanced cell tracking technologies will allow real-time monitoring of the localization, migration, and differentiation of transplanted MSCs, which is crucial for understanding the behavior of MSCs in vivo.

Finally, the formulation and implementation of treatment strategies may necessitate interdisciplinary collaboration and technological innovation involving nephrologists, immunologists, and cell therapy specialists. Key to the widespread application of MSCs in renal therapy will be optimizing MSC production and quality control, enhancing their stability and efficiency in vivo, and developing more personalized therapies. These advances are anticipated to improve treatment methods for kidney diseases, enhance patients' quality of life, and ensure that each patient receives the maximum benefit from MSC therapies.

## Figures and Tables

**Table 1:** Surface markers of MSCs and characterization of related heterogeneity.

Marker category	Marker name	Function/relevance
Surface marker	CD105 (Endoglin)	Related to cell adhesion, migration, and angiogenesis [5, 73]
Surface marker	CD73 (5′-Nucleotidase)	Involved in cell signaling and immunoregulation [5, 17]
Surface marker	CD90 (Thy-1)	Associated with cell adhesion, immune regulation, and cell survival [5, 73]
Surface marker	CD44	Linked to cell migration, tumorigenesis, and cell signaling [5, 74]
Surface marker	STRO-1	Related to self-renewal and pluripotency of cells [73]
Surface marker	CD146 (MCAM)	Involved in cell adhesion, migration, and angiogenesis [5, 72]
Surface marker	CD106 (VCAM-1)	Related to cell adhesion and immune response [5, 17]
Surface marker	CD166 (ALCAM)	Associated with cell adhesion and migration [73]
Surface marker	CD54 (ICAM-1)	Involved in cell adhesion and immune response [5, 73]
Negative marker	CD45	Excludes cells of hematopoietic origin [17]
Negative marker	CD34	Excludes endothelial cells and hematopoietic progenitors [17]
Negative marker	CD14 or CD11b	Excludes macrophages and monocytes [17]
Negative marker	HLA-DR	Excludes specific immune cells [17]
Functional marker	SSEA-4	Related to pluripotency and self-renewal capabilities [73]
Functional marker	Oct-4, Sox2, and Nanog	Maintains stem cell characteristics of MSCs [5, 73]
Functional marker	CD200	Immunomodulatory function [75]
Other marker	ALP (alkaline phosphatase)	Related to osteogenic differentiation [74]
Other marker	CD271 (NGF receptor)	Associated with neural regeneration [72]

## Data Availability

The data presented in this review are from the cited references.

## References

[1] (2021). Acute Kidney Injury. *Nature Reviews Disease Primers*.

[2] Hoste E., Kellum J. A., Selby N. M. (2018). Global Epidemiology and Outcomes of Acute Kidney Injury. *Nature Reviews Nephrology*.

[3] Fraser S. D. S., Roderick P. J. (2019). Kidney Disease in the Global Burden of Disease Study 2017. *Nature Reviews Nephrology*.

[4] Ronco C., Bellomo R., Kellum J. A. (2019). Acute Kidney Injury. *The Lancet*.

[5] Viswanathan S., Shi Y., Galipeau J. (2019). Mesenchymal Stem versus Stromal Cells: International Society for Cell & Gene Therapy (ISCT(R)) Mesenchymal Stromal Cell Committee Position Statement on Nomenclature. *Cytotherapy*.

[6] Yun C. W., Lee S. H. (2019). Potential and Therapeutic Efficacy of Cell-Based Therapy Using Mesenchymal Stem Cells for Acute/Chronic Kidney Disease. *International Journal of Molecular Sciences*.

[7] Zhang F., Wang C., Wen X. (2020). Mesenchymal Stem Cells Alleviate Rat Diabetic Nephropathy by Suppressing CD103^+^ DCs-Mediated CD8^+^ T Cell Responses. *Journal of Cellular and Molecular Medicine*.

[8] Yuan Y., Yuan L., Li L. (2021). Mitochondrial Transfer From Mesenchymal Stem Cells to Macrophages Restricts Inflammation and Alleviates Kidney Injury in Diabetic Nephropathy Mice via PGC-1*α* Activation. *Stem Cells*.

[9] Luo Y., Guo J., Zhang P. (2021). Mesenchymal Stem Cell Protects Injured Renal Tubular Epithelial Cells by Regulating mTOR-Mediated Th17/Treg Axis. *Frontiers in Immunology*.

[10] Fan Y., Chen H., Huang Z., Zheng H., Zhou J. (2020). Emerging Role of miRNAs in Renal Fibrosis. *RNA Biology*.

[11] Joo H. S., Suh J. H., Lee H. J., Bang E. S., Lee J. M. (2020). Current Knowledge and Future Perspectives on Mesenchymal Stem Cell-Derived Exosomes as a New Therapeutic Agent. *International Journal of Molecular Sciences*.

[12] Wu L., Rong C., Zhou Q. (2021). Bone Marrow Mesenchymal Stem Cells Ameliorate Cisplatin-Induced Renal Fibrosis via miR-146a-5p/Tfdp2 Axis in Renal Tubular Epithelial Cells. *Frontiers in Immunology*.

[13] Yea J.-H., Yoon Y. M., Lee J. H., Yun C. W., Lee S. H. (2021). Exosomes Isolated From Melatonin-Stimulated Mesenchymal Stem Cells Improve Kidney Function by Regulating Inflammation and Fibrosis in a Chronic Kidney Disease Mouse Model. *Journal of Tissue Engineering*.

[14] Zhang Y., Le X., Zheng S. (2022). MicroRNA-146a-5p-Modified Human Umbilical Cord Mesenchymal Stem Cells Enhance Protection Against Diabetic Nephropathy in Rats Through Facilitating M2 Macrophage Polarization. *Stem Cell Research & Therapy*.

[15] Usunier B., Brossard C., L’Homme B. (2021). HGF and TSG-6 Released by Mesenchymal Stem Cells Attenuate Colon Radiation-Induced Fibrosis. *International Journal of Molecular Sciences*.

[16] Li Y., Ricardo S. D., Samuel C. S. (2022). Enhancing the Therapeutic Potential of Mesenchymal Stromal Cell-Based Therapies With an Anti-Fibrotic Agent for the Treatment of Chronic Kidney Disease. *International Journal of Molecular Sciences*.

[17] Dominici M., Le Blanc K., Mueller I. (2006). Minimal Criteria for Defining Multipotent Mesenchymal Stromal Cells. The International Society for Cellular Therapy position statement. *Cytotherapy*.

[18] Spees J. L., Lee R. H., Gregory C. A. (2016). Mechanisms of Mesenchymal Stem/Stromal Cell Function. *Stem Cell Research & Therapy*.

[19] Mohammadalipour A., Dumbali S. P., Wenzel P. L. (2020). Mitochondrial Transfer and Regulators of Mesenchymal Stromal Cell Function and Therapeutic Efficacy. *Frontiers in Cell and Developmental Biology*.

[20] Abumaree M. H., Abomaray F. M., Alshabibi M. A., AlAskar A. S., Kalionis B. (2017). Immunomodulatory Properties of Human Placental Mesenchymal Stem/Stromal Cells. *Placenta*.

[21] Abbaszadeh H., Ghorbani F., Derakhshani M. (2020). Regenerative Potential of Wharton’s Jelly-Derived Mesenchymal Stem Cells: A New Horizon of Stem Cell Therapy. *Journal of Cellular Physiology*.

[22] Zhang S., Wang J. Y., Li B., Yin F., Liu H. (2021). Single-Cell Transcriptome Analysis of Uncultured Human Umbilical Cord Mesenchymal Stem Cells. *Stem Cell Research & Therapy*.

[23] Qu R., He K., Fan T. (2021). Single-Cell Transcriptomic Sequencing Analyses of Cell Heterogeneity During Osteogenesis of Human Adipose-Derived Mesenchymal Stem Cells. *Stem Cells*.

[24] Wang Z., Chai C., Wang R. (2021). Single-Cell Transcriptome Atlas of Human Mesenchymal Stem Cells Exploring Cellular Heterogeneity. *Clinical and Translational Medicine*.

[25] Chen P., Tang S., Li M. (2023). Single-Cell and Spatial Transcriptomics Decodes Wharton’s Jelly-Derived Mesenchymal Stem Cells Heterogeneity and a Subpopulation With Wound Repair Signatures. *Advanced Science*.

[26] Kim M., Bae Y. K., Um S. (2020). A Small-Sized Population of Human Umbilical Cord Blood-Derived Mesenchymal Stem Cells Shows High Stemness Properties and Therapeutic Benefit. *Stem Cells International*.

[27] Wang Z., Li X., Yang J. (2021). Single-Cell RNA Sequencing Deconvolutes the In Vivo Heterogeneity of Human Bone Marrow-Derived Mesenchymal Stem Cells. *International Journal of Biological Sciences*.

[28] Klinkhammer B. M., Kramann R., Mallau M. (2014). Mesenchymal Stem Cells From Rats With Chronic Kidney Disease Exhibit Premature Senescence and Loss of Regenerative Potential. *PLoS ONE*.

[29] Chen H., Wen X., Liu S. (2023). Dissecting Heterogeneity Reveals a Unique BAMBI^high^ MFGE8^high^ Subpopulation of Human UC-MSCs. *Advanced Science*.

[30] Guerrero E. N., Vega S., Fu C., De León R., Beltran D., Solis M. A. (2021). Increased Proliferation and Differentiation Capacity of Placenta-Derived Mesenchymal Stem Cells From Women of Median Maternal Age Correlates With Telomere Shortening. *Aging*.

[31] Tammaro A., Kers J., Scantlebery A. M. L., Florquin S. (2020). Metabolic Flexibility and Innate Immunity in Renal Ischemia Reperfusion Injury: The Fine Balance Between Adaptive Repair and Tissue Degeneration. *Frontiers in Immunology*.

[32] Doke T., Abedini A., Aldridge D. L. (2022). Single-Cell Analysis Identifies the Interaction of Altered Renal Tubules With Basophils Orchestrating Kidney Fibrosis. *Nature Immunology*.

[33] Cao J. Y., Wang B., Tang T. T. (2021). Exosomal miR-125b-5p Deriving From Mesenchymal Stem Cells Promotes Tubular Repair by Suppression of p53 in Ischemic Acute Kidney Injury. *Theranostics*.

[34] Wang W., Zhang M., Ren X. (2023). Single-Cell Dissection of Cellular and Molecular Features Underlying Mesenchymal Stem Cell Therapy in Ischemic Acute Kidney Injury. *Molecular Therapy*.

[35] Zhang J., Su R., Wang Y. (2024). Protective Effect of Small Extracellular Vesicles (EVs) Derived From ACE2-Modified Human Umbilical Cord Mesenchymal Stem Cells against Renal Ischemia-Reperfusion Injury. *Nephrology*.

[36] Jo S. Y., Cho H. J., Kim T. M. (2023). Fenoldopam Mesylate Enhances the Survival of Mesenchymal Stem Cells Under Oxidative Stress and Increases the Therapeutic Function in Acute Kidney Injury. *Cell Transplantation*.

[37] Abdelrahman S. A., Raafat N., Abdelaal G. M. M., Aal S. M. A. (2023). Electric Field-Directed Migration of Mesenchymal Stem Cells Enhances Their Therapeutic Potential on Cisplatin-Induced Acute Nephrotoxicity in Rats. *Naunyn-Schmiedeberg’s Archives of Pharmacology*.

[38] Huang H., Qian M., Liu Y. (2023). Genetically Engineered Mesenchymal Stem Cells as a Nitric Oxide Reservoir for Acute Kidney Injury Therapy. *eLife*.

[39] Hegde M., Singh A. K., Kannan S., Kolkundkar U., Seetharam R. N. (2024). Therapeutic Applications of Engineered Mesenchymal Stromal Cells for Enhanced Angiogenesis in Cardiac and Cerebral Ischemia. *Stem Cell Reviews and Reports*.

[40] Nagaishi K., Mizue Y., Chikenji T. (2016). Mesenchymal Stem Cell Therapy Ameliorates Diabetic Nephropathy via the Paracrine Effect of Renal Trophic Factors including Exosomes. *Scientific Reports*.

[41] Xiang E., Han B., Zhang Q. (2020). Human Umbilical Cord-Derived Mesenchymal Stem Cells Prevent the Progression of Early Diabetic Nephropathy Through Inhibiting Inflammation and Fibrosis. *Stem Cell Research & Therapy*.

[42] KDIGO (2020). Clinical Practice Guideline for Diabetes Management in Chronic Kidney Disease. *Kidney International*.

[43] Sun J., Zhao F., Zhang W., Lv J., Lv J., Yin A. (2018). BMSCs and miR-124a Ameliorated Diabetic Nephropathy via Inhibiting Notch Signalling Pathway. *Journal of Cellular and Molecular Medicine*.

[44] Jin J., Shi Y., Gong J. (2019). Exosome Secreted From Adipose-Derived Stem Cells Attenuates Diabetic Nephropathy by Promoting Autophagy Flux and Inhibiting Apoptosis in Podocyte. *Stem Cell Research & Therapy*.

[45] Wang L., Qing L., Liu H. (2017). Mesenchymal Stromal Cells Ameliorate Oxidative Stress-Induced Islet Endothelium Apoptosis and Functional Impairment via Wnt4-*β*-Catenin Signaling. *Stem Cell Research & Therapy*.

[46] Lee S. E., Jang J. E., Kim H. S. (2019). Mesenchymal Stem Cells Prevent the Progression of Diabetic Nephropathy by Improving Mitochondrial Function in Tubular Epithelial Cells. *Experimental & Molecular Medicine*.

[47] Lin W., Li H. Y., Yang Q. (2021). Administration of Mesenchymal Stem Cells in Diabetic Kidney Disease: A Systematic Review and Meta-Analysis. *Stem Cell Research & Therapy*.

[48] Hickson L. J., Abedalqader T., Ben-Bernard G. (2021). A Systematic Review and Meta-Analysis of Cell-Based Interventions in Experimental Diabetic Kidney Disease. *Stem Cells Translational Medicine*.

[49] Li W., Chen W., Sun L. (2021). An Update for Mesenchymal Stem Cell Therapy in Lupus Nephritis. *Kidney Diseases*.

[50] Li J., Luo M., Li B. (2022). Immunomodulatory Activity of Mesenchymal Stem Cells in Lupus Nephritis: Advances and Applications. *Frontiers in Immunology*.

[51] Chua A., Guo D., Tan J. C. (2023). Intraperitoneally Delivered Umbilical Cord Lining Mesenchymal Stromal Cells Improve Survival and Kidney Function in Murine Lupus via Myeloid Pathway Targeting. *International Journal of Molecular Sciences*.

[52] Liang J., Zhang H., Hua B. (2010). Allogenic Mesenchymal Stem Cells Transplantation in Refractory Systemic Lupus Erythematosus: A Pilot Clinical Study. *Annals of the Rheumatic Diseases*.

[53] Wang D., Li J., Zhang Y. (2014). Umbilical Cord Mesenchymal Stem Cell Transplantation in Active and Refractory Systemic Lupus Erythematosus: A Multicenter Clinical Study. *Arthritis Research & Therapy*.

[54] Ranjbar A., Hassanzadeh H., Jahandoust F. (2022). Allogeneic Adipose-Derived Mesenchymal Stromal Cell Transplantation for Refractory Lupus Nephritis: Results of a Phase I Clinical Trial. *Current Research in Translational Medicine*.

[55] Yuan X., Qin X., Wang D. (2019). Mesenchymal Stem Cell Therapy Induces FLT3L and CD1c+ Dendritic Cells in Systemic Lupus Erythematosus Patients. *Nature Communications*.

[56] Makhlough A., Shekarchian S., Moghadasali R. (2018). Bone Marrow-Mesenchymal Stromal Cell Infusion in Patients With Chronic Kidney Disease: A Safety Study With 18 Months of Follow-up. *Cytotherapy*.

[57] Abumoawad A., Saad A., Ferguson C. M. (2020). In a Phase 1a Escalating Clinical Trial, Autologous Mesenchymal Stem Cell Infusion for Renovascular Disease Increases Blood Flow and the Glomerular Filtration Rate While Reducing Inflammatory Biomarkers and Blood Pressure. *Kidney International*.

[58] Erpicum P., Weekers L., Detry O. (2019). Infusion of Third-Party Mesenchymal Stromal Cells after Kidney Transplantation: A Phase I-II, Open-Label, Clinical Study. *Kidney International*.

[59] Wei Y., Chen X., Zhang H. (2021). Efficacy and Safety of Bone Marrow-Derived Mesenchymal Stem Cells for Chronic Antibody-Mediated Rejection After Kidney Transplantation- A Single-Arm, Two-Dosing-Regimen, Phase I/II Study. *Frontiers in Immunology*.

[60] Veceric-Haler Z., Sever M., Kojc N. (2022). Autologous Mesenchymal Stem Cells for Treatment of Chronic Active Antibody-Mediated Kidney Graft Rejection: Report of the Phase I/II Clinical Trial Case Series. *Transplant International*.

[61] Wu Z., Xu X., Cai J. (2022). Prevention of Chronic Diabetic Complications in Type 1 Diabetes by Co-Transplantation of Umbilical Cord Mesenchymal Stromal Cells and Autologous Bone Marrow: A Pilot Randomized Controlled Open-Label Clinical Study With 8-Year Follow-up. *Cytotherapy*.

[62] Perico N., Remuzzi G., Griffin M. D. (2023). Safety and Preliminary Efficacy of Mesenchymal Stromal Cell (ORBCEL-M) Therapy in Diabetic Kidney Disease: A Randomized Clinical Trial (NEPHSTROM). *Journal of the American Society of Nephrology*.

[63] Swaminathan M., Stafford-Smith M., Chertow G. M. (2018). Allogeneic Mesenchymal Stem Cells for Treatment of AKI After Cardiac Surgery. *Journal of the American Society of Nephrology*.

[64] Swaminathan M., Kopyt N., Atta M. G. (2021). Pharmacological Effects of Ex Vivo Mesenchymal Stem Cell Immunotherapy in Patients With Acute Kidney Injury and Underlying Systemic Inflammation. *Stem Cells Translational Medicine*.

[65] Yang Y., Gao J., Wang S. (2022). Efficacy of Umbilical Cord Mesenchymal Stem Cell Transfusion for the Treatment of Severe AKI: A Protocol for a Randomised Controlled Trial. *BMJ Open*.

[66] Badve S. V., Palmer S. C., Hawley C. M., Pascoe E. M., Strippoli G. F. M., Johnson D. W. (2016). Glomerular Filtration Rate Decline as a Surrogate End Point in Kidney Disease Progression Trials. *Nephrology Dialysis Transplantation*.

[67] Levey A. S., Inker L. A., Matsushita K. (2014). GFR Decline as an End Point for Clinical Trials in CKD: A Scientific Workshop Sponsored by the National Kidney Foundation and the US Food and Drug Administration. *American Journal of Kidney Diseases*.

[68] Perkovic V., Koitka-Weber A., Cooper M. E. (2020). Choice of Endpoint in Kidney Outcome Trials: Considerations From the EMPA-REG OUTCOME® Trial. *Nephrology Dialysis Transplantation*.

[69] Merkhan M. M., Shephard M. T., Forsyth N. R. (2021). Physoxia Alters Human Mesenchymal Stem Cell Secretome. *Journal of Tissue Engineering*.

[70] Vieira C. P., McCarrel T. M., Grant M. B. (2021). Novel Methods to Mobilize, Isolate, and Expand Mesenchymal Stem Cells. *International Journal of Molecular Sciences*.

[71] de Matos B. M., Robert A. W., Stimamiglio M. A., Correa A. (2022). Pluripotent-Derived Mesenchymal Stem/Stromal Cells: An Overview of the Derivation Protocol Efficacies and the Differences Among the Derived Cells. *Stem Cell Reviews and Reports*.

[72] Sasse S., Skorska A., Lux C. A., Steinhoff G., David R., Gaebel R. (2020). Angiogenic Potential of Bone Marrow Derived CD133^+^ and CD271^+^ Intramyocardial Stem Cell Trans- Plantation Post MI. *Cells*.

[73] Ullah I., Subbarao R. B., Rho G. J. (2015). Human Mesenchymal Stem Cells - Current Trends and Future Prospective. *Bioscience Reports*.

[74] Jia Y., Wang A., Zhao B. (2022). An Optimized Method for Obtaining Clinical-Grade Specific Cell Subpopulations From Human Umbilical Cord-Derived Mesenchymal Stem Cells. *Cell Proliferation*.

[75] Imboden S., Liu X., Lee B. S., Payne M. C., Hsieh C.-J., Lin N. Y. C. (2021). Investigating Heterogeneities of Live Mesenchymal Stromal Cells Using AI-Based Label-Free Imaging. *Scientific Reports*.

[76] Wang Z., Chen H., Wang P. (2022). Site-Specific Integration of TRAIL in iPSC-Derived Mesenchymal Stem Cells for Targeted Cancer Therapy. *Stem Cells Translational Medicine*.

